# DNA Barcodes and Species Distribution Models Evaluate Threats of Global Climate Changes to Genetic Diversity: A Case Study from *Nanorana parkeri* (Anura: Dicroglossidae)

**DOI:** 10.1371/journal.pone.0103899

**Published:** 2014-08-05

**Authors:** Wei-wei Zhou, Bao-lin Zhang, Hong-man Chen, Jie-qiong Jin, Jun-xiao Yang, Yun-yu Wang, Ke Jiang, Robert W. Murphy, Ya-ping Zhang, Jing Che

**Affiliations:** 1 State Key Laboratory of Genetic Resources and Evolution, and Yunnan Laboratory of Molecular Biology of Domestic Animals, Kunming Institute of Zoology, Chinese Academy of Sciences, Kunming, China; 2 Laboratory for Conservation and Utilization of Bio-resources, Yunnan University, Kunming, China; 3 Centre for Biodiversity and Conservation Biology, Royal Ontario Museum, Toronto, Ontario, Canada; Biodiversity Insitute of Ontario - University of Guelph, Canada

## Abstract

Anthropogenic global climate changes are one of the greatest threats to biodiversity. Distribution modeling can predict the effects of climate changes and potentially their effects on genetic diversity. DNA barcoding quickly identifies patterns of genetic diversity. As a case study, we use DNA barcodes and distribution models to predict threats under climate changes in the frog *Nanorana parkeri*, which is endemic to the Qinghai-Tibetan Plateau. Barcoding identifies major lineages W and E. Lineage W has a single origin in a refugium and Lineage E derives from three refugia. All refugia locate in river valleys and each greatly contributes to the current level of intraspecific genetic diversity. Species distribution models suggest that global climate changes will greatly influence *N. parkeri*, especially in the level of genetic diversity, because two former refugia will fail to provide suitable habitat. Our pipeline provides a novel application of DNA barcoding and has important implications for the conservation of biodiversity in southern areas of the Qinghai-Tibetan Plateau.

## Introduction

Climatic changes influence organisms and an understanding of how this occurs is important for conservation. More than one line of evidence documents the impact anthropogenic global climate change (GCC) exerts on organisms [1]. Explorations into how past climate changes influenced organisms may serve to predict future impacts of GCC. Genetic diversity is important in conservation because higher levels maintain the evolutionary potential of species. However, the distribution of genetic diversity is often uneven across the range of a species and many factors may contribute to this. Environmental changes during glacial-interglacial cycling in the Quaternary is one of the most important historical drivers of genetic patterns [2], [3]. For example, by retaining suitable habitat over several glacial cycles, refugia hold higher levels of genetic diversity compared with recently occupied areas [2], [3]. Refugia also drive genetic distinctiveness within species owing to providing long-term geographic isolation. A clear understanding a species' evolutionary history and its drivers is important for planning conservation [4].

Genetic analyses form the cornerstone of conservation planning, especially in defining objective evolutionarily significant units (ESUs) and management units (MUs) [5]. DNA barcoding [6], which uses a short, universal genetic marker (*COI* in eukaryotes) to identify matrilines and species, may serve to efficiently identify ESUs and MUs.

Species distribution models (SDMs) also provide information useful for conservation planning [7]–[9]. Comparisons between SDMs for current and the Last Glacial Maximum (LGM) may provide a chance to explore the impacts of past climate changes to organisms. As an extension of this application, SDMs can compare the distributions of current and future habitats. This allows assessments of the risk of local extirpation and extinction caused by future habitat degradation. Whereas both DNA barcoding and SDMs provide valuable insights, their synthesis may serve to evaluate threats of GCC to organisms.

The Qinghai-Tibetan Plateau (QTP), which covers more than 2.5 million km^2^ and has an average elevation of about 4000 m above sea level [10], is the largest and highest plateau on Earth. Unlike North America and Europe, no unified ice sheet formed on the QTP during the LGM, yet its environment changed substantially. The average temperature was from 6°C to 9°C lower than today and precipitation decreased by from 30% to 70% [11].

In addition to climatic drivers, geographic features also play a role in the formation of patterns of genetic diversity. In the southern QTP, two mountains stretch from east to west and the Yarlung Zangpo River (YZR; Brahmaputra River) (Fig. S2) occurs between them. The Nianqingtanggula Mountains (NM; Nyainqentanglha Mountains) in the east and Gangdisi Mountains (GM; Kailas Range) in the west form the northern mountains and the Himalayas form the southern boundary. Several rivers flow southward across the latter. The complex geography may have offered refugia during dramatic Quaternary climatic changes. Analyses of matrilineal dispersal are likely to reveal such history. Driven by the GCC, future severe temperature changes of the QTP may exceed those of lower elevations [12]. Thus, the GCC may impose enormous impacts on organisms living in the QTP. We assess this possibility by synthesizing DNA barcoding and SDMs analyses.

Because of their relatively low mobility and physiological requirements, amphibians are sensitive to environmental changes, especially temperature and precipitation. Further, amphibians retain high-resolution signals of historical responses to environmental perturbations [13]. The environment on the QTP is harsh for amphibians; suitable environment is rare except in the eastern and southern edges. A previous study on *Rana kukunoris*, a common frog on eastern QTP, revealed a unique genetic pattern compared to other taxa in the region [14]. Comparative amphibian studies from the southern QTP will yield insights into how environmental changes affect the biota fauna of the QTP.

*Nanorana parkeri*, a median-sized frog belonging to the family Dicroglossidae, is endemic to southern and southeastern Tibet. It occurs at elevations ranging from 2800 to 5000 m in valleys of the YZR and north of the NM [15]. Climate changes during LGM greatly affected this area. Our study evaluates samples from across the entire range of *N. parkeri*. Using the species as model system, we test the effectiveness of a pipeline that combines DNA barcoding and SDMs to identify future threats to genetic diversity in the face of GCC.

## Materials and Methods

### Ethics Statement

All the species included in our study (Table S1) are not endangered or protected species according to the “Law of the People's Republic of China on the Protection of Wildlife” and “Regulations for the Implementation of the People's Republic of China on the Protection of terrestrial Wildlife” (State Council Decree [1992] No. 13). The permission for field work in four major management areas (39 localities) including Xigaze, Lhasa, Nyingchi and Qamdo was issued by the Forestry Department of Xizang [Tibet] Autonomous Region, China. Local people of the Forestry Bureaus involved and helped during the whole survey. Totally, 549 tissue samples including toe tips, muscle, livers, tadpoles and egg masses were obtained, following the Animal Use Protocols approved by the Animal Care and Ethics Committee of the Kunming Institute of Zoology, Chinese Academy of Sciences. For egg masses, we separated 5–10 eggs from egg mass and stored them in 95% alcohol after removing egg jelly. For adult, within each locality only five individuals were euthanized using clove oil firstly. Following euthanization, tissues dissected from adult specimens were preserved in 95% ethanol. More other adults were just cut two toe tips and then released. Tadpoles were also euthanized using clove oil firstly, then stored in 95% alcohol after removing gut. Table S1 lists our samplings information, including species name, locality, GPS coordinates, and accession nos. in Genbank etc.

### Population sampling and laboratory techniques

**Figure 1:**
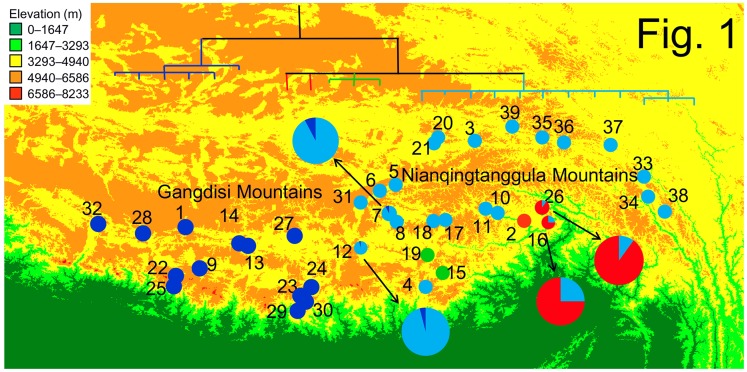
Sampling sites and the maternal genealogy based on mtDNA sequence data. Site numbers refer to Table S1. Colors of the matrilines match those of samplings localities on the map. Four localities containing two matrilines each are show in larger pie figures.

Our samplings covered the entire documented distribution range of *N. parkeri* (Fig. 1, Table S1). One individual of *N. pleskei*, *N. ventripunctata* and *N. liebigii* was used as an outgroup representative [16], [17].

We extracted genomic DNA using standard phenol–chloroform extraction protocol [18]. Partial sequences of cytochrome c oxidase subunit I (*COI*) were sequenced for all individuals using universal primers [19]. PCR products were purified and used as the template DNA for cycle sequencing reactions performed using BigDye Terminator Cycle Sequencing Kit (v.2.0, Applied Biosystems, Foster City, USA) and an ABI PRISM 3730 DNA Analyzer.

### Sequence alignment and phylogenetic analyses

Nucleotide sequences were checked by eye using LASERGENE 7.0 and aligned using CLUSTALX 1.81 [20] with default parameters. Subsequently, the aligned sequences were checked and optimized in MEGA 4.0 [21]. Identical haplotypes for mtDNA were collapsed using DNASP 5.10 [22]. The overall value of nucleotide diversity (π) and haplotype diversity (H) were also estimated using DNASP.

Phylogenetic analyses of the *COI* data were conducted using Bayesian inference (BI), maximum likelihood (ML) and maximum parsimony (MP). BI analyses were performed using MrBayes 3.1.2 [23]. We tested three different partition strategies based on codon positions (no partition; 1+2, 3; and 1, 2, 3). The best strategy was chosen based on the Bayes factor test [24] (Table 1), as it represented a robust method testing partitioning strategies [25]. Nucleotide substitution models were selected for each data partition using the Akaike information criterion in MrModeltest v2.3 [26]. BI analyses for each partition strategy were run 3 million generations while sampling trees every 1000 generations. The first 50% of the sampled trees were discarded as burn-in. The final analyses employing the best partition strategy were run for 10 million generations. AWTY [27] was used to confirm satisfactory convergence of the topological split frequencies. MP analyses were implemented using PAUP* 4.0b10 [28]. Heuristic searches with TBR were executed for 1000 random pseudoreplicates with all characters treated as unordered and equally weighted. Bootstrap analyses were conducted using 1000 replicates to assess nodal reliabilities. ML analyses were conducted using RaxML 7.0.4 [29] and the GTR+I+G model was implemented for each data partition.

**Table 1:** Bayes factor of each partition strategy.

Partition Strategy	Ln(BF)	Standard Deviation
no partition	−1885.375	0.134
1+2, 3	−1844.969	0.14
1, 2, 3	−1885.375	0.12

2lnBF (H_1_–H_0_) >10 was treated as decisive support for each hypothesis.

We built a median-joining network (MJN) using NETWORK 4.5 [30] to visualize the frequencies of the haplotypes. To remove excessive links and median vectors, we used the MP option [31].

### Divergence time estimation

Different demographic and molecular clock models were compared using path sampling and stepping-stone sampling (Table 2) [32]. Employing the best models, time to most recent common ancestor was estimated using BEAST 1.7.5 [33]. Due to the absence of a reliable fossil record and an established substitution rate of *COI* for *N. parkeri*, we used the divergence time between *N. parkeri* and *N. pleskei* (8.9±2.7 Ma) [16] as a secondary calibration point. Model selection and partition scheme were the same as used in the BI analyses. The final analyses involved two independent runs of 30 million generations each, while sampling trees every 1000^th^ generation. Burn-in and convergence of the chains were determined with Tracer 1.5 [34]. The measures of effective sample sizes were used to determine the Bayesian statistical significance of each parameter.

**Table 2:** Results of demographic model selection.

	strick		lognormal relaxed clock	
	PS	SS	PS	SS
constant	−1553.29	−1553.31	−1553.33	−1553.34
exponential	−1556.44	−1556.53	−1556.24	−1556.27
logistic	−1557.29	−1557.36	−1555.73	−1555.77
expansion	−1554.42	−1554.45	−1555.05	−1555.07
bayesian skyline	−1544.51	−1544.56	−1545.42	−1545.46

PS: path sampling; SS: stepping-stone sampling.

### Population structure and demographic analyses

We explored population structure and genetic diversity landscape using SPADS 1.0 [35]. Groups of populations were defined as for SAMOVA [36]. We explored values of K (number of groups) ranging from 2 to 10 with 100 simulated annealing processes. The optimum value of K was identified by exploring the behavior of the indices F_CT_ and F_SC_. Spatial patterns of genetic diversity based on allelic richness (Ar) and π across the landscape were explored using the GDivPAL function in SPADS.

To test for the influences of mountains and rivers on the genetic structure of *N. parkeri*, we measured the population structure by three independent analyses of molecular variance (AMOVA) [37]: populations north and south of the YZR; populations north of NM, west of the boundary of GM and NM, and the remaining ones; and four groups according the results of the SAMOVA. Analyses were performed using Arlequin 3.5 [38] and significance was assessed by 10000 permutations.

We investigated past changes of effective population size using the neutral test and mismatch distributions. Tajima's *D* [39] and Fu's *Fs* statistics [40] with 10000 coalescent simulations were calculated using Arlequin. Pairwise mismatch distributions [41] were calculated for each group in Arlequin. The expected distribution under a model of sudden demographical expansion was generated using 10000 permutations. The significance of deviations from this model was tested using the sum of squared deviation (SSD) and raggedness index (Rag). All analyses were performed for each lineage separately because population subdivisions could have masked the effects of expansions.

### Species distribution modeling

We inferred the potential geographic range of *N. parkeri* using the maximum entropy model implemented in MAXENT 3.3.3 [42], [43]. Environmental variables from the WorldClim database with resolutions of 2.5 arc-minutes [44] were downloaded as environmental layers. Because of the controversy about whether correlated variables should be removed or not, we used all of the 19 bioclimatic layers [45]. All layers were cropped to span from 83°E to 99°E and from 26°N to 33°N.

Random null distributions were built to test for the significance of our SDM. For this test, we built a new SDM using 39 random points (as same number as our sampling localities). This process was repeated 100 times and the areas under the curves (AUCs) were used as null distributions [46].

Assuming niche conservatism over time [47]–[49], we predicted the former distribution of *N. parkeri* by projecting our model on LGM climatic layers. Predicted distributions during the LGM were generated by downloading both the community climate system model (CCSM) [50] and model for interdisciplinary research on climate (MIROC) [51] from the WorldClim database. To predict the influences of GCC to this species, we also projected the model to climate data of the 2080 s based on MIROC model under the A1b scenario. In this way, we predicted the distribution changes in future.

## Results

### Sequence information

We obtained 549 partial *COI* sequences from 39 localities of *N. parkeri*, plus one sequence each from *N. pleskei*, *N. ventripunctata* and *N. liebigii*. The fragment consisted of 539 base pairs, of which 35 positions exhibited variation and 27 were potentially parsimony informative, resulting in 23 haplotypes in *N. parkeri*. All sequences were deposited in GenBank (Table S1). The overall value of π was 0.01746±0.00055 and H was 0.678±0.016.

### Genealogical analyses of *COI*

Bayes factor test showed a preference for the partition strategy of 1+2, 3 (Table 1). The best fit substitution model for the first and second codon was HKY and GTR+G model was the best fit for third codon.

**Figure 2:**
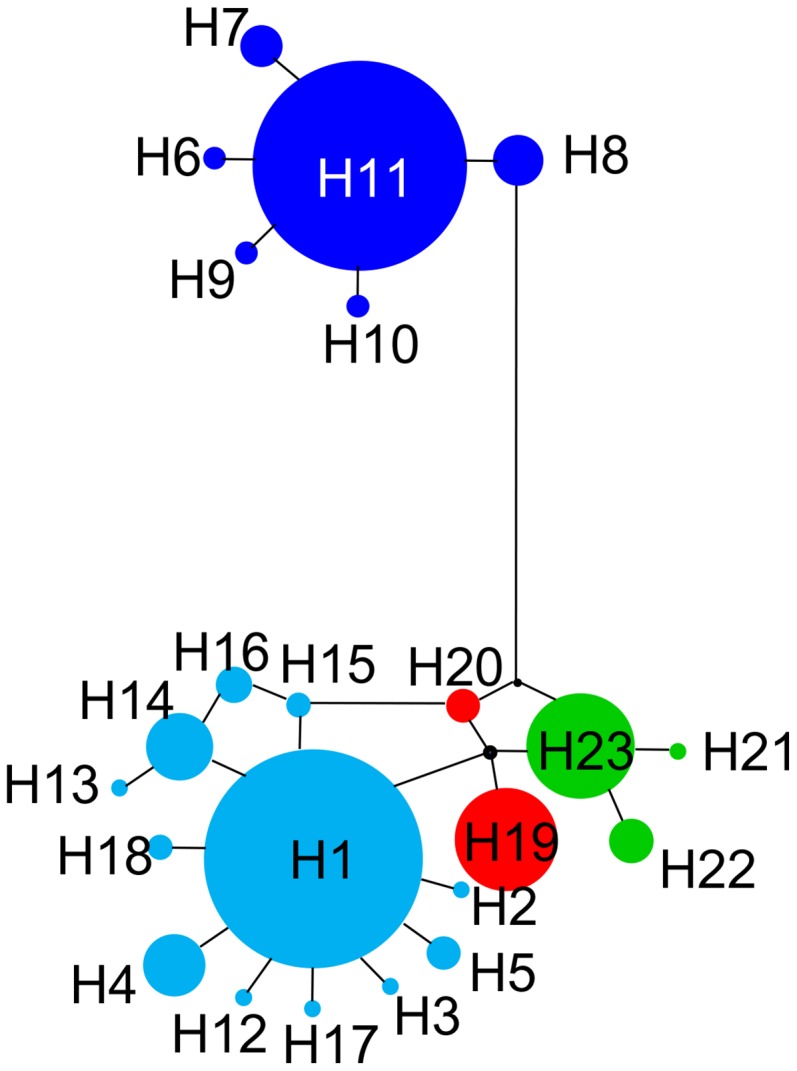
The median-joining network of *COI* haplotypes. Colors correspond to maternal lineages in Figure 1.

Matrilineal genealogies obtained from BI, ML and MP analyses were nearly identical (Fig. 1 & Fig. S1) and all methods recovered lineages East (E) and West (W). The boundary of GM and NM separated these two lineages. Lineage W was comprised of 6 haplotypes, which had no clear structure. Lineage E was comprised 17 haplotypes of which 12 formed highly supported sublineage E1. The MJN depicted patterns similar to those of the gene tree (Fig. 2). Haplotypes H1 and H11, which were more common than the other haplotypes, occupied central positions.

### Divergence time estimates

**Figure 3:**
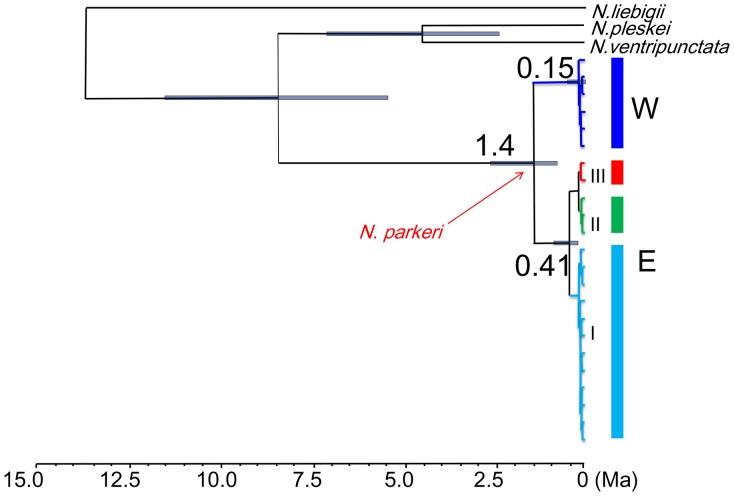
Estimates of divergence times obtained using BEAST 1.7.5.

Results of model comparisons were shown in Table 2. Both path sampling and stepping-stone sampling suggested that the BSP demographic model under strict clock outperformed the alternative models. The average divergence time estimations were shown in Fig. 3. Lineages E and W diverged about 1.4 Ma (95% CI: 0.6–2.4 Ma). Lineage E diverged about 0.41 Ma (95% CI: 0.15–0.81 Ma) and the radiation of Lineage W happened about 0.15 Ma (95% CI: 0.05–0.32 Ma).

### Genetic structure and demographic history

**Figure 4:**
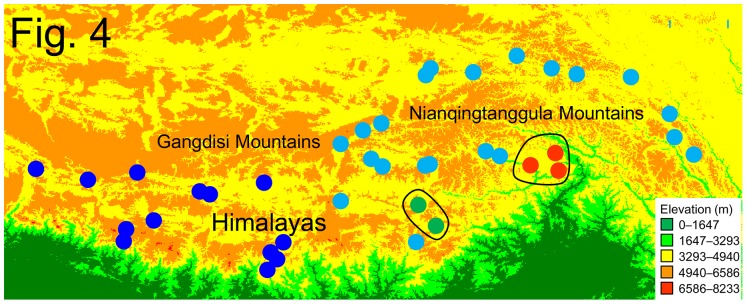
Results of SAMOVA. Different colors represent different groups. A solid line encircles two groups of Lineage E from microrefugia.

**Figure 5:**
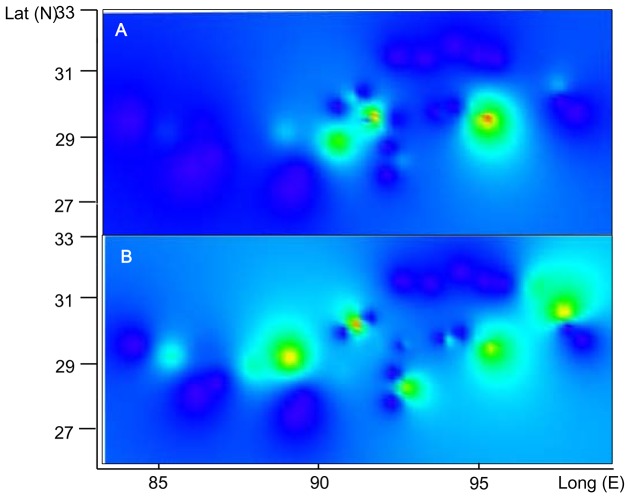
Genetic diversity across the range of *Nanorana parkeri*. X axis represents longitude and the Y axis latitude. A: π, pattern of nucleotide diversity; B: Ar, pattern of allelic richness.

Results of population clustering were illustrated in Figure 4. F_CT_ plateaued when K was 4 (Table S2). Samples from Lineage W grouped together (Fig. 4, dark blue). Lineage E contained 3 groups, of which populations in the southern edges of the QTP formed two small groups (localities 2, 16 and 26; and locality 15 and 19); the remaining localities comprised the third group (Fig. 4, light blue). Spatial patterns of genetic diversity based on Ar and π were shown in Figure 5 and listed in Table S3. Several peaks indicated high levels of genetic diversity across the distribution of *N. parkeri*. This was congruent with the pattern of lineage divergence.

When forming two groups based on the YZR, among-group diversity accounted for 16.04% of the overall variation and among-populations within groups accounted for 80.76%. Dividing populations according to the mountains, among-group diversity accounted for 87.90% of the overall variation and among-populations within groups accounted for 9.67%. Finally, after dividing populations based on results of the SAMOVA, among-group diversity accounted for 96.88% of the overall variation and among-populations within groups accounted for 0.97% (Table 3).

**Table 3:** Results of AMOVA.

Group compositions	Among group	Among populations	Within populations	ΦSC	ΦST	ΦCT
By River	16.04%	80.76%	3.20%	0.96183	0.96795	0.16039
By Mountain	87.90%	9.67%	2.42%	0.79973	0.97578	0.87905
By SAMOVA	96.88%	0.97%	2.16%	0.30944	0.97844	0.96877

**Figure 6:**
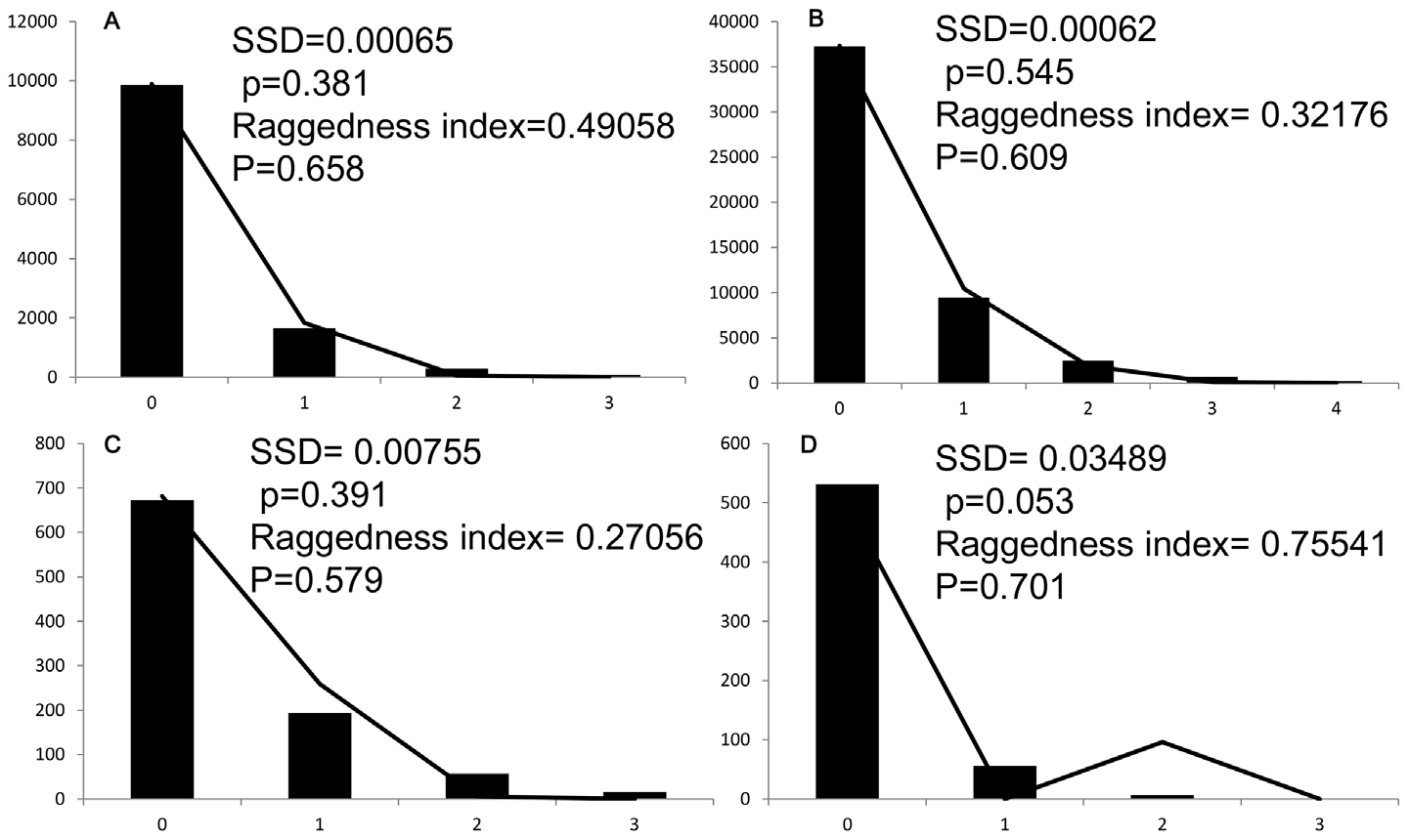
Results of the mismatch distribution for *Nanorana parkeri*. Bars indicate simulated mismatch distributions and lines denote the observed mismatch distributions. A: lineage W; B: sublineage E1; C: sublineage E2; D: sublineage E3.

For Lineage W, the values of Tajima's *D* and Fu's *Fs* were significantly negative (Table 4), which indicated population expansions. The hypothesis of sudden expansion was not rejected by mismatch distribution analyses (Fig. 6) as the SSD and Rag were insignificant (P_SSD_ = 0.381 and P_RAG_ = 0.658). In sublineage E1, significantly negative values of both Tajima's *D* and Fu's *Fs* supported population expansions. The mismatch distribution analyses did not reject the sudden expansion model (P_SSD_ = 0.545 and P_RAG_ = 0.609). In sublineages E2, Tajima's *D* and Fu's *F*s were negative, but insignificant. In sublineage E3, Tajima's *D* was negative but Fu's *Fs* was positive, yet all values were not significant. The null hypothesis of sudden expansion model was not rejected by mismatch distribution analyses.

**Table 4:** Statistics of neutrality tests for each lineage and sublineage.

	Tajima's D	P value	Fu's Fs	P value
W	−1.62115	0.0044	−6.41652	0.0005
E1	−1.80395	0.0033	−14.10103	0
E2	−0.69425	0.2176	−0.77698	0.1858
E3	−0.65715	0.2279	0.92064	0.5177

### Species distribution modeling

The AUCs from random data ranged from 0.605 to 0.800 (0.733±0.033; Table S4). For the SDMs of *N. parkeri*, the AUC of our data was 0.904 and this was significant better than that of a random model (P<0.001).

**Figure 7:**
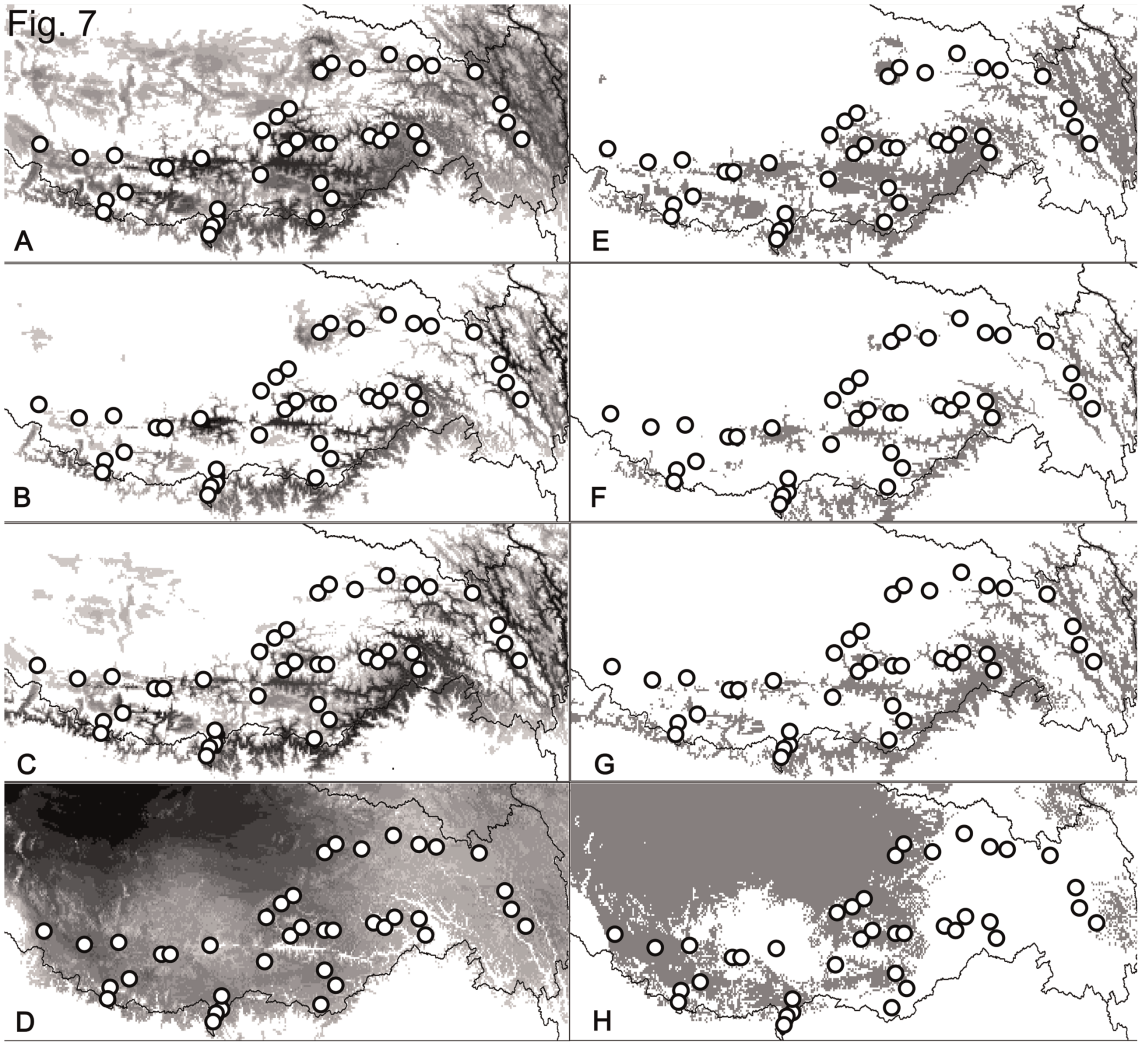
The predicted distribution of *Nanorana parkeri*. A: predicted distribution based on current data; B: distribution during the LGM based on MIROC model; C: distribution during the LGM based on CCSM model; D: the predicted distribution in the 2080 s under GCC; E–H: predicted distributions above the threshold for models A–D, respectively.

The MIROC model predicted that the distribution of *N. parkeri* at the LGM was much smaller than that of today (Fig. 7). The results based on CCSM model suggested a similar pattern, but with larger areas than those from MIROC model. Both models suggested range fluctuations after the LGM in northern, high altitude areas. The boundary area between GM and NM, where lineages W and E divided, has been unsuitable for *N. parkeri* since the LGM. The predicted distribution under GCC assuming the A1b scenario was shown in Figure 7. *Nanorana parkeri* is predicted to have a larger distribution in the northwestern QTP. However, the species' distribution will contract in the southeastern QTP.

## Discussion

### Lineage divergence and ESUs

ESUs, which can be determined by genetic differentiation at neutral markers caused by isolation, are important when considering conservation actions. Our study builds on the framework for delineating ESUs of *N. parkeri* by reconstructing the patterns of lineage divergence and its drivers. Our analyses suggest the recognition of ESUs that correspond to lineages W, E1, E2 and E3. Our research demonstrates that *COI* can quickly delineate ESUs.

The gene trees and MJN depict a clear east-west split for *N. parkeri*. SDMs suggest that unsuitable habitat between the GM and NM, both now and during the LGM, drives this pattern. The boundary is the demarcation line of two QTP climatic zones that are largely congruent with the 400 mm precipitation line. East of this line, the region is semi-humid, and to the west semi-arid [52]. Similar pattern was also found in *Hippophae tibetana* [53], [54].

Geographic features, such as rivers or mountains, appear to contribute little to the patterns of genetic divergence. The YZR, which would impose a north-south split, does not drive population divergence. The AMOVA explains only 16.04% of the genetic variation in this scenario. Elevations of NM range from 5000 m to 6000 m, but no substantial genetic divergence distinguishes frogs in south and north of NM. Lineages E and W are separated by the boundary of GM and NM. However, habitat barriers and environmental differences more likely generate this pattern than geographical features. Two observations support this hypothesis. First, populations of lineages W and E both occur south of the GM and NM. Second, *N. parkeri* occurs along the YZR valley, which flows across the boundary and connects lineages W and E.

The pattern of genetic divergence within each major lineage differs. The 6 haplotypes in Lineage W do not yield a clear pattern (Fig. 1). The network depicts a star-like haplogroup (Fig. 2). SAMOVA also suggests populations from W comprise a single group. In contrast, Lineage E differs in having three groups (Figs. 1, 2). Results from SAMOVA present the same pattern. Most northern populations form a group as do frogs from localities 15, 19 (E2) and localities 2, 16, 26 (E3). The difference in patterns between E and W may owe to topography. The intricate topography of the East Himalayas, which is a global biodiversity hotspot, provides suitable habitats and generates barriers that limit dispersal for Lineage E. Other species exhibit a similar pattern; populations in the fringes or southern slope of Himalayas are genetically different from those on the QTP [54], [55]. Expansion and contraction of distribution ranges caused by climate changes provided opportunity for populations divergence in QTP [54]–[57].

The genetic pattern within *N. parkeri* suggests recognition of four ESUs, W, E1, E2 and E3. Certainly gene flow occurs within and between lineages E and W, as lineages or sublineages mix at localities 8, 12, 16 and 26. However, matrilineal uniqueness occurs in populations far from the boundary.

### Glacial refugia and genetic diversity of *N. parkeri*

Refugia in the QTP prevented extinction during the LGM. In doing so, they conserved high levels of genetic diversity. In contrast, genetic diversity in recently occupied areas is much lower because of founder effects [2], [3]. The identification of refugia is an important part of conservation because these areas preserve genetic diversity. Synthesizing DNA barcoding and SDMs, we quickly and effectively identify refugia for *N. parkeri*. Populations in refugia retain higher levels of diversity than population in newly occupied places (Fig. 5 and Table S3).

The multiple refugia hypothesis corresponds with patterns of mtDNA divergence. The matrilineal genealogy, MJN and SAMOVA analyses indicate the absence of genetic structure within Lineage W. Thus, Lineage W likely originates from a single refugium. The SDMs indicate the presence of suitable habitat in the river valley near locality 27 (Fig. 7) and the area retains a much higher level of genetic diversity (Fig. 5) than other sites. The neutral test and mismatch distribution analyses clearly detect a population expansion in Lineage W.

Our analyses identify several refugia in Lineage E. Sublineage E1 may originate from a northern refugium near localities 7 and 8, which occur in the valley at the confluence of the Lhasa River and the YZR (Fig. 7). During the LGM the suitable environment harbored a high level of genetic diversity. The absence of genealogical structure in sublineage E1 is congruent with a sudden population expansion, as are significantly negative values of Tajima's *D* and Fu's *Fs*, and the mismatch distribution analyses. Further, our analyses identify two microrefugia in the river valleys along southern edges of the QTP. Localities 2, 16 and 26, which are in the YZR Valley near Nyingchi and Medog, seemingly constitute a microrefugium. The other microrefugium consists of localities 15 and 19, which are also in a river valley. This river flows southwards across the Himalayas. The lower elevations of the river valleys make them less susceptible to the influences montane glaciers. River valleys in the Himalayas, especially those connected with southern slopes, appear to offer microrefugia.

Populations from sampling localities north of the NM also retain a high level of genetic diversity (Fig. 5). Private haplotypes H4 (locality 38), H2 and H3 (locality 33), and H5 (locality 37) occur here (Table S1). The SDMs suggest that suitable habitat existed in northern areas of NM. This area appears to harbor another microrefugium. However, these private haplotypes fail to cluster together and the most common haplotype in the area is H1 (Fig. 2 & Table S1). Thus, we cannot reject the hypothesis that these private haplotypes originated during population expansions after the LGM.

### Potential threats of GCC to *N. parkeri*

Our study suggests that the combination of DNA barcoding and SDMs can detect threats of GCC to the survival of species. This pipeline facilitates conservation. SDMs predict that suitable habitat for *N. parkeri* may experience great shifts in the near future. Whereas the Northwest QTP will offer suitable habitat for *N. parkeri*, the Southeast QTP will become unsuitable. Although suitable habitat will experience an overall expansion, populations in the Southeast QTP may experience sharp decreases in population size or become extirpated. Given that amphibians have poor dispersal abilities, the latter scenario may be an unfortunate consequence of GCC.

Our pipeline suggests that many populations of Lineage E may suffer from developing unsuitable habitats. The microrefugium near Nyingchi and Medog will become unsuitable under this prediction (Fig. 7). Fortunately, suitable habitat may persist in the two other refugia. Thus, genetic variation will decrease yet all may not be lost. For Lineage W, suitable habitat will disappear near the refugium at locality 27. Loss of this refugium will greatly decrease the amount of genetic diversity.

Our analyses suggest that genetic diversity of *N. parkeri* may greatly decrease. Although this facilitates effective conservation planning, we urge caution. Our SDMs for the future assume A1b scenarios, which involve rapid global economic growth with a balance of fossil and non-fossil energy sources. Each of possible scenarios yields different predictions about the future climate. All scenarios require constant adjustment according to global economic conditions. The complex landscape in the southern QTP may supply suitable microhabitats for *N. parkeri* which cannot be detected by our SDMs. Accordingly, we suggest the urgent development of an effective monitoring program, especially for populations in refugia that may lose suitable habitats.

## Conclusion

DNA barcoding detects the genetic structure of *N. parkeri* and serves to define ESUs. Our analyses recover major lineages E and W, which separate at the boundary of the GM and NM. Habitat barriers and environmental differences, combined with geographic features, are the drivers of genetic divergences. Lineage E contains three parts: E1, E2 and E3. Thus our analyses define four ESUs that correspond to matrilines W, E1, E2 and E3. Genetic and environmental data identify four historical refugia, each of which corresponds to a lineage or sublineage. Lineage W originates from a refugium in river valley near locality 27. Most populations of Lineage E originate from a refugium in the river valley near Lhasa. Two microrefugia occur in river valleys along the Himalayas. Our analyses detect a population mixture after the LGM in some localities but these occurrences do not influence our designations of ESUs. Our study highlights the importance of valleys along the Himalayas for biodiversity conservation. Based on climate models under GCC, we predict the potential distribution changes and threats to genetic diversity of *N. parkeri*. Our pipeline, which combines DNA barcoding and SDMs, is an effective approach in conservation.

## Supporting Information

Figure S1 **Matrilineal genealogy of** ***Nanorana parkeri*** **based on BI analyses of** ***COI*** **sequence data.** Bootstrap proportions ≥70% and Bayesian posterior probabilities ≥95% were treated as strongly supported (▾) and bootstrap proportions ≥70% and Bayesian posterior probabilities ≥90% were treated as being moderately supported. Bootstrap proportions<70% and Bayesian posterior probabilities <90% were treated as being unsupported (*).
(TIF)

Figure S2 **River systems in the southern QTP.**
(TIF)

Table S1 **Detailed information for specimens included in this study.**
(XLSX)

Table S2 **Values of F_CT_, F_ST_ and F_SC_ based on population groups suggested by SAMOVA.**
(XLSX)

Table S3 **Values of π and Ar for populations from each locality.**
(XLSX)

Table S4 **Detailed results of AUC for random and real data.**
(XLSX)
