# Supplementary figures and images for: DNA Barcodes and Species Distribution Models Evaluate Threats of Global Climate Changes to Genetic Diversity: A Case Study from Nanorana parkeri (Anura: Dicroglossidae)
Source: PLoS One. 2014 Aug 5;9(8):e103899. doi: 10.1371/journal.pone.0103899 (PMC4122371; doi:10.1371/journal.pone.0103899)

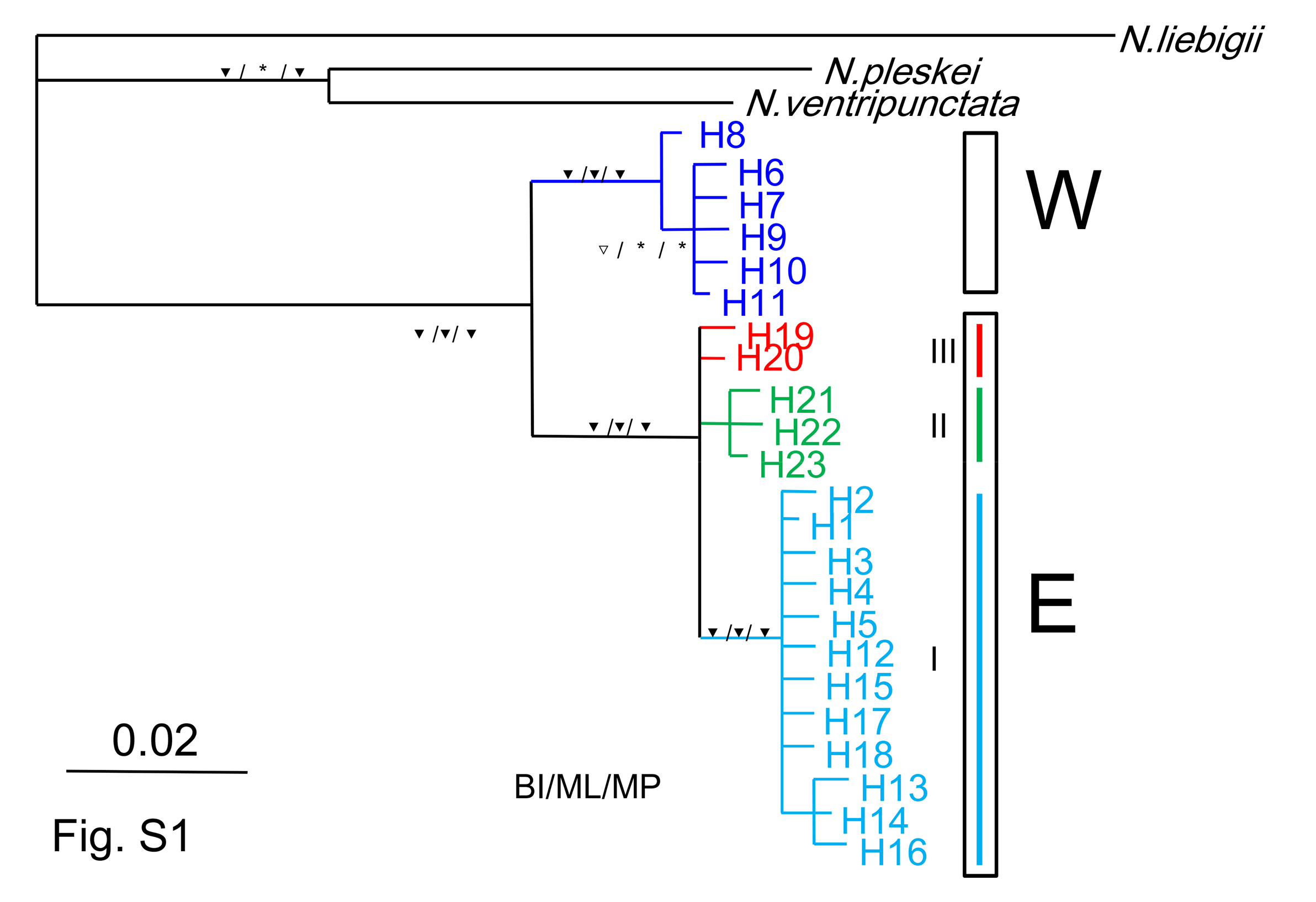

Supplement: Figure S1 — Matrilineal genealogy of Nanorana parkeri based on BI analyses of COI sequence data. Bootstrap proportions ≥70% and Bayesian posterior probabilities ≥95% were treated as strongly supported (▾) and bootstrap proportions ≥70% and Bayesian posterior probabilities ≥90% were treated as being moderately supported. Bootstrap proportions<70% and Bayesian posterior probabilities <90% were treated as being unsupported (*). (TIF) [file pone.0103899.s001.tif]

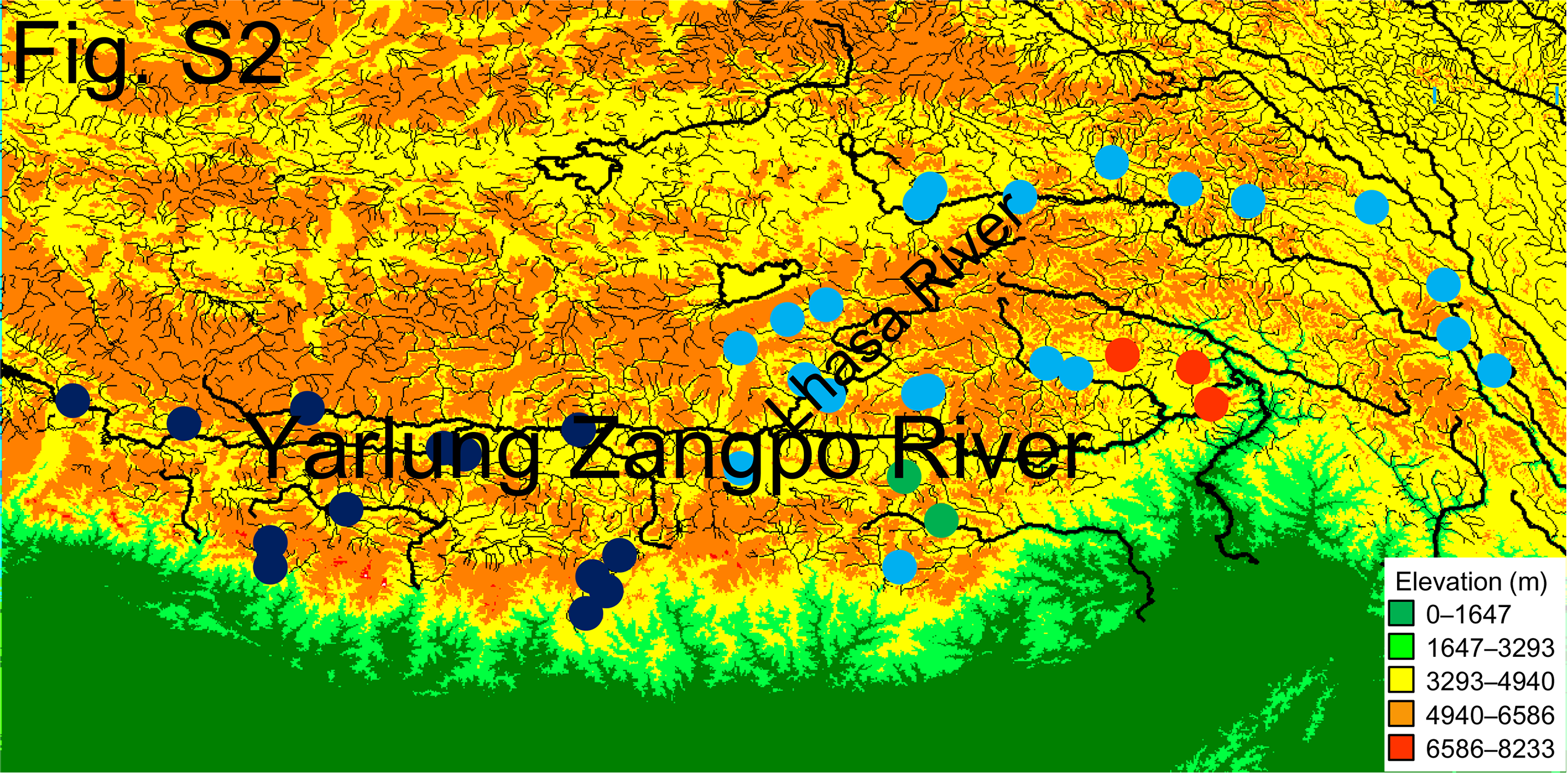

Supplement: Figure S2 — River systems in the southern QTP. (TIF) [file pone.0103899.s002.tif]
